# Maternal Impressions

**Published:** 1878-06-20

**Authors:** 


### MATERNAL IMPRESSIONS.

"Mrs. H., mother of several healthy children, was severely shocked during the pregnancy referred to in this report by a sad accident to her husband, and which afterward proved fatal.

"To make the case more intelligible, I will first relate the accident referred to. Mr. H., a pump-maker, was engaged in a well, at the depth of thirty-five feet, staying a pump, when the stone walls suddenly gave way. The stones forming a partial arch over his head prevented his being instantly crushed. After sixteen hours of anxious, weary labor—his voice, faint and indistinct, being audible all the time—he was found still living, with his arms and legs clasped around the pump-log; a position into which he sprang, as he afterward stated, when he felt the stones moving. When taken out, cold and numb, his feet were turned inward, as in the act of climbing. Two stones had pressed upon him: one, upon the head, left a contusion; the other, upon the lumbar region of the spine, produced a slough. He lived only five days after the accident. During this time he was very restless, but much relieved when some person leaned over him so that he could clasp his hands around them. Mrs. H., six months advanced in pregnancy, was present at the rescue, and nursed her husband almost without intermission up to the time of his death. Three months afterward she gave birth to a deformed infant, the abnormalties of which bore a striking resemblance to the condition and marks on the father produced by the accident in the well. Its feet were turned inward, with double talipes varus. On the side of the head was an ecchymosis, and in the lumbar region of the spine a wound differing from an ordinary spina bifida, in there being no abnormal fluid in the subarachnoid space, and besides the spinal processes and laminæ of the part, all the structures external to the membranes of the cord were deficient. The cord, of normal size, was visible through the membranes. The wounds on the head and spine corresponded to those referred to on the father; more especially the latter, as a slough when removed leaves exposed the normal structures underneath. The child lived five days, the same length of time as the father lived after the accident. Another and the most remarkable coincidence was that the child resembled the father in not resting only when some one held its hands firmly grasped. The latter circumstance I could not believe until I saw unmistakable evidence of it. As I entered the room one day, the child was sleeping quietly, the nurse holding its hands inclosed in her own. She mentioned to me the peculiarity, and as I expressed myself as being doubtful of the fact, she quietly and gently relaxed her hold. No sooner done than the child screamed as if in great distress, and as soon as she seized them again it became calm and quiet, and remained so while the hands were held."—*Dr. MacKay, in Canada Lancet.*

### IODOFORM IN DYSENTERY.

A writer in *Med. Brief* says that iodoform is an efficient remedy in chronic dysentery. It is also strongly recommended as a remedy for typhoid fever. Dose, 1 gr. three times per day in pill.

